# The RhoGDIβ-Rac1-CARD9 Signaling Module Mediates Islet β-Cell Dysfunction Under Chronic Hyperglycemia

**DOI:** 10.3390/cells14141046

**Published:** 2025-07-09

**Authors:** Anjaneyulu Kowluru, Jie-Mei Wang

**Affiliations:** 1 Biomedical Research Service, John D. Dingell VA Medical Center, Detroit, MI 48201, USA; 2 Department of Pharmaceutical Sciences, Eugene Applebaum College of Pharmacy and Health Sciences, Wayne State University, Detroit, MI 48201, USA; jiemei.wang@wayne.edu

**Keywords:** islet beta cell, Rac1, RhoGDIβ, CARD9, beta cell dysfunction, metabolic stress, type 2 diabetes

## Abstract

Small (monomeric) GTP-binding proteins (smgs; Cdc42 and Rac1) play requisite roles in islet beta cell function, including glucose-stimulated insulin secretion. In addition, emerging evidence suggests that sustained (constitutive) activation of smgs (e.g., Rac1) culminates in the genesis of islet beta cell dysfunction under the duress of chronic hyperglycemia. It is noteworthy that functions (i.e., activation–deactivation) of smgs in many cells, including the islet beta cell, have been shown to be under the regulatory control of at least three factors, namely the guanine nucleotide exchange factors (GEFs), the GTPase-activating proteins (GAPs), and the GDP-dissociation inhibitors (GDIs). The overall objective of this review is to highlight our current understanding of the regulatory roles of the RhoGDIβ-Rac1-CARD9 signalome in the pathology of beta cell dysfunction under chronic hyperglycemic stress. For brevity, this review is structured by an overview of smgs and their regulatory proteins/factors in the beta cell, followed by a discussion of potential roles of the RhoGDIβ-Rac1-CARD9 axis in the onset of cellular dysfunction under the duress of metabolic stress. Overall conclusions, potential knowledge gaps, and opportunities for future research in this field of islet biology are highlighted in the last section.

## 1. Introduction

Diabesity (obesity and type 2 diabetes) is now considered the biggest global epidemic [1]. According to the recent estimates by the International Diabetes Federation (IDF), nearly 589 million adults (i.e., 20–79 years age group) are living with diabetes, which represents a staggering 11.1% of the world’s population within this age group. Unfortunately, the total number of adults with diabetes is expected to increase to 853 million by 2050. More alarmingly, an estimated 635 million adults are living with impaired glucose tolerance [2]. These data warrant an immediate need for not only understanding the mechanisms underlying the metabolic dysregulation and demise of the islet β-cell but also for increased efforts to develop novel therapeutics for halting β-cell defects and the onset of diabetes and its associated complications.

Experimental and clinical evidence suggests that, during the early stages of the onset of type 2 diabetes mellitus (T2DM), the β-cell works diligently to increase the biosynthesis and release of insulin to compensate for insulin resistance in the target peripheral tissues. As a consequence, the “over-worked” islet β-cell undergoes metabolic exhaustion, leading to the diminished production and release of insulin, culminating in β-cell failure, loss of functional beta cell mass, and the onset of T2DM. Despite a high number of bench-to-bedside investigations in the field to understand the pathophysiology of T2DM, putative mechanisms that dictate islet β-cell dysfunction and demise in T2DM remain elusive to date [3,4].

This review is written with the objective of highlighting our current understanding of potential defects in small GTP-binding protein (smgs)-controlled signaling pathways in the islet beta cell exposed to chronic metabolic stress. For brevity, this article is organized in the following sequence: At the outset, a brief review of existing knowledge about smgs and their regulatory factors, namely guanine nucleotide exchange factors (GEFs), GDP-dissociation inhibitors (GDIs), and GTPase-activating proteins (GAPS), in islet function in health and metabolic stress is provided. This is followed by an overview of the available evidence on the potential impact of cellular stress on RhoGDIs, specifically, the RhoGDIβ, at the onset of cellular dysregulation under a variety of stress conditions, including metabolic stress. Please note that RhoGDIβ is also referred to as LyGDI, GDID4, RhoGDI2 or ARHGDIB in the literature. For brevity, RhoGDIβ is used in all studies that highlighted the alternate names of this RhoGDI. Subsequent to this is a brief summary of available evidence suggesting the potential involvement of the RhoGDIβ-Rac1 signaling module in the onset of cellular dysfunction under the duress of hyperglycemic stress. The last section highlights conclusions, potential knowledge gaps, and opportunities for future research in this field of islet biology.

## 2. Smgs and Their Regulatory Factors in Islet Function

Glucose-induced (physiological) insulin secretion (GSIS) from the beta cell involves a variety of metabolic and cationic events. Some of these include the intracellular generation of hydrophobic (e.g., diacyl glycerol) and hydrophilic (cAMP and inositol triphosphates) second messenger molecules, as well as an increase in intracellular calcium [5,6,7,8,9,10]. In addition to the increase in adenine nucleotides (ATP), an increase in the generation of guanine nucleotides (GTPs) is implicated in the stimulus–secretion coupling of GSIS. Seminal investigations by Metz et al. [11,12,13] have demonstrated critical permissive roles for GTP in GSIS and suggested that intracellular GTP is requisite for the activation of one (or more) smgs in the beta cell.

**Figure 1:**
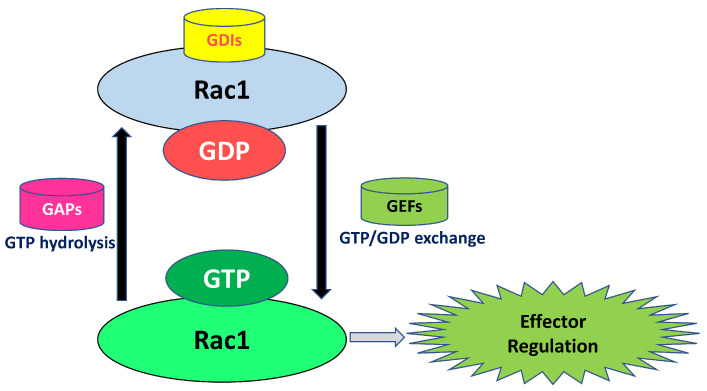
Schematic representation of Rac1 GTP-hydrolytic cycle. Under basal conditions, GDP-bound Rac1 (inactive conformation) remains complexed with the RhoGDI in the cytosolic compartment. Under conditions of cellular activation requisite for optimal physiological insulin secretion (e.g., exposure to stimulatory glucose), GDP-bound Rac1/RhoGDI complex dissociates, paving the way for the activation of Rac1 by a variety of GEFs, which are involved in the exchange of GDP for GTP, resulting in the activation of Rac1.GTP. Following the activation of appropriate effectors and facilitation of actin cytoskeletal rearrangements, which are requisite for GSIS, the active form of Rac1 (GTP-bound) is converted back to its inactive (GDP-bound) conformation by the GTPase-activating proteins (GAPs), leading to the complexation of GDP-bound Rac1 with the RhoGDI, thus completing the Rac1 activation–deactivation cycle. Despite our current understanding of regulatory roles of GDIs and GEFs in Rac1-mediated events leading to insulin secretion, very little is known with regard to the identity and roles of putative GAPs that control Rac1 deactivation on various regulatory factors involved in acute and sustained activation of Rac1 under normal and metabolic stress conditions, respectively (see text for additional description). Abbreviations: GDIs: GDP-dissociation inhibitors; GEFs: guanine nucleotide exchange factors; GAPs: GTPase-activating proteins; GDP: guanosine diphosphate; GTP: guanosine triphosphate.

At least two major classes of G proteins have been implicated in the cascade of events leading to GSIS. The first group consists of heterotrimeric G proteins, which comprise three subunits (α/β/γ subunits) of differing molecular weights [14,15,16]. They are implicated in the coupling of various G protein-coupled receptors (GPCRs) to their intracellular effector proteins, including adenylate cyclase, phosphodiesterase, or phospholipases. The second group is the monomeric smgs (Arf6, Rho, Cdc42, and Rac1); these signaling proteins partake in protein sorting, vesicular trafficking, cytoskeletal rearrangement, and fusion of secretory vesicles with the membrane [17,18]. Available evidence implicates both classes of G proteins in islet β-cell function, including cell proliferation and insulin secretion [18]. Mechanistically, both trimeric G proteins and smgs undergo activation–deactivation cycles (GTP-hydrolytic cycle) during their activation and regulation of various effector proteins. In the case of smgs (e.g., Rac1; focus of this review), the cycling between their GDP-bound (inactive) and GTP-bound (active) conformation is facilitated by specific regulatory proteins/factors (Figure 1). At least three classes of such regulatory molecules/factors have been identified thus far [19,20,21]. GEFs facilitate the exchange of GDP for GTP, resulting in their active GTP-bound conformation. Previous studies have identified a number of GEFs for smgs in clonal beta cells, normal rodent islets, and human islets. These include T-cell lymphoma invasion and metastasis 1 (Tiam1), Vav guanine nucleotide exchange factor 2 (Vav2), ARF nucleotide binding site opener (ARNO), β-PIX (also known as Arhgef7 or Cool1), IQ motif containing GTPase-activating protein 1 (IQGAP1), and exchange protein directly activated by cAMP (Epac) [17,18,22]. GAPs promote the hydrolysis of GTP bound to smgs to GDP by augmenting the intrinsic guanosine triphosphatase (GTPase) functions of smgs to complete their GTP-hydrolytic cycle. Published studies have identified a variety of GAPs in the islet beta cells, including AS160, ARHGAP21, Stard13, and TBC1D1 [18,23,24,25]. Recent investigations by Wang et al. have defined novel roles for the Adaptor Protein–Phosphotyrosine Interacting with PH Domain and Leucine Zipper 2 (APPL2) as a modulator of Rac1GAP in the signaling events leading to GSIS, thus highlighting the involvement of additional regulatory molecules in Rac1 activation–deactivation cycle [26]. Lastly, GDIs significantly contribute to smg functions. For example, they have been shown to prevent the association of smgs with their relevant membranous sites by masking and limiting the prenylation of smgs at their C-terminal cysteines. In addition, they are reported to impact the functions of GAPs and GEFs, thereby regulating the activation of smgs. Lastly, they are implicated in the inhibition of proteasomal degradation of smgs, and the extraction of GTP-bound smgs from their respective membranes for spatiotemporal regulation of their function [27,28,29]. It is noteworthy that studies from the Thurmond laboratory have identified caveolin-1, a scaffolding protein, as one of the GDIs responsible for the functional regulation of Cdc42 in the signaling events leading to GSIS [30]

While a large number of GEFs (~82) and GAPs (~70) have been identified, only three GDIs (RhoGDIα, RhoGDIβ, and RhoGDIγ) are expressed in mammalian cells [27,28,31]. RhoGDIα (also known as RhoGDI1) is ubiquitously expressed and partakes in the functional regulation of smgs (RhoA, Cdc42, and Rac1). Previous studies have implicated RhoGDIα in physiological insulin secretion [32]. RhoGDIβ (also known as LyGDI, GDID4, RhoGDI2, or ARHGDIB) was originally believed to be expressed only in hematopoietic cells. However, emerging evidence suggests that it is expressed in non-hematopoietic cells (keratinocytes, fibroblasts, amnion cells, and lung and colon cancer cells) [28,31,33,34,35,36]. It is noteworthy that even though RhoGDIβ shares 67% amino acid identity with RhoGDIα, the former is less able to regulate GDP/GTP exchange or to promote the dissociation of smgs from the target cellular compartments (e.g., plasma membrane). Functionally, RhoGDIβ has been shown to participate in actin cytoskeletal organization, immune response, vascular remodeling, and cellular apoptosis [27,37]. We recently provided the first evidence for the expression of RhoGDIβ in INS-1 832/13 cells, MIN6 cells, rodent islets, and human islets [38,39]. Data accrued in these investigations have also provided fresh insights into the regulatory roles of RhoGDIβ in the islet beta cell function in health and metabolic stress (focus of this article). Lastly, RhoGDIγ, the third GDI, is expressed in the lung, brain, and testis. Recent evidence suggests that it is expressed in clonal beta (INS-1 832/13 and MIN6) cells, as well as normal rodent islets and human islets [38,39]. Evidence on other cells suggests that RhoG, an smg, is under the regulatory control of RhoGDIγ, while our data on beta cells, although with inconclusive evidence, suggest a potential crosstalk between RhoG and RhoGDIγ [40]. The next section highlights the impact of cellular stress on RhoGDIβ expression and function in various cell types, including the islet beta cell.

## 3. Impact of Cellular Stress on the Structure and Function of RhoGDIβ

### 3.1. Evidence in Other Cells

Several earlier investigations have demonstrated a significant increase in the expression of RhoGDIβ in a variety of pathological conditions, including cancer [31,41]. Interestingly, however, the expression of RhoGDIβ can vary among different forms of cancer. Moreover, published evidence suggests the caspase-3-mediated degradation of RhoGDIβ at Asp19 (ΔN(1–19)LyGDI) in cells under the duress of exposure to apoptotic stimuli, such as ionizing radiation [42], anti-Fas [43], anti-IgM antibody [44], TNFα [45], staurosporine [46], or taxol [47]. From a mechanistic standpoint, it has been demonstrated that the proteolytic cleavage of RhoGDIβ at Asp19 leads to the translocation of the truncated form of RhoGDIβ to the nuclear compartment for propagation of signals necessary for cell apoptosis. Investigations in BJAB Burkitt-like lymphoma cells, by Essman et al., have revealed that RhoGDIβ, but not RhoGDIα, is cleaved by caspase-3 during drug-induced apoptosis [47]. Using two-dimensional gel electrophoresis, mass spectrometry, and immunoblotting techniques, Choi and coworkers provided additional insights into the nuclear association of the truncated product of RhoGDIβ [48]. A shift in the isoelectric point of Δ19-RhoGDIβ to a more basic pH allows this truncated product to remain in the nucleus. Furthermore, the fusion of the p53 nuclear export signaling sequence to Δ19-RhoGDIβ significantly prevented the ability of the latter to promote apoptosis. The overexpression of Δ19-RhoGDIβ markedly increased the susceptibility of cells to apoptosis following incubation with staurosporine. It was concluded that the caspase-3-mediated degradation of RhoGDIβ and associated generation of Δ19-RhoGDIβ leads to accelerated cellular apoptosis [48].

Krieser and Eastman have also demonstrated proteolytic degradation of RhoGDIβ under conditions of apoptosis [46]. Zinc and a variety of phosphatase inhibitors, including okadaic acid, calyculin A, and cantharidin, as well as z-VAD-fmk, a known inhibitor of caspases, were reported to significantly inhibit RhoGDIβ and cell death. Interestingly, subcellular distribution experiments demonstrated a nuclear association of the truncated product of RhoGDIβ, not the full-length RhoGDIβ, which remained largely in the cytosolic compartment. Lastly, site-specific mutation at the proteolytic site of RhoGDIβ prevented its hydrolysis without eliciting any significant effects on the degree of apoptosis induced by staurosporine. These findings have led to the conclusion that the caspase-3-mediated cleavage of RhoGDIβ and subsequent translocation of the degradation product might not be contributing to cell demise [46]. It is noteworthy that RhoGDIβ also undergoes caspase-mediated degradation at Asp55, resulting in the generation of ΔN(1–55) RhoGDIβ. Data from studies in inflammatory leukocytes implicated ΔN(1–55) RhoGDIβ in the activation of Rho family smgs (e.g., Rac1 and Cdc42) due to its inability to inhibit GDP dissociation [49]. Together, the above studies support the overall postulation that RhoGDIβ undergoes caspase-3-mediated degradation at Asp19 and Asp55 and that the truncated products elicit regulatory effects on cell function. Available evidence on the roles of RhoGDIβ in beta cell function in health and metabolic stress is reviewed below.

### 3.2. Evidence in Pancreatic Islet Beta Cells

Data accrued in extant investigations have suggested key roles for RhoGDIs, specifically, RhoGDIα, in GSIS [32]. However, it is only recently that the potential roles of RhoGDIβ and RhoGDIγ have been examined in the context of islet β-cell function under normal health conditions and metabolic stress. For example, recent investigations from our laboratory have examined putative roles for RhoGDIβ in glucose-induced Rac1 activation and insulin secretion in insulin-secreting INS-1 832/13 cells [38]. We reported the expression of all three forms of RhoGDI in clonal beta cells, rodent islets, and human islets. The siRNA-mediated depletion of RhoGDIβ significantly attenuated the activation of Rac1 induced acutely by glucose. RhoGDIβ depletion had minimal impact on GSIS, even under conditions of a significant reduction in glucose-induced activation of Rac1. It was concluded that RhoGDIβ might contribute to the signaling steps involved in glucose-induced activation of Rac1 but may not directly contribute to insulin secretion [38].

Along these lines, we have recently investigated the metabolic fate of RhoGDIβ in pancreatic beta cells following exposure to chronic hyperglycemic stress [39]. Therein, we asked whether the exposure of pancreatic β-cells to chronic hyperglycemic stress leads to caspase-3-induced degradation of RhoGDIβ. We noted a significant increase in the expression of full-length RhoGDIβ in INS-1 832/13 cells following exposure to hyperglycemic conditions. A significant increase in the abundance of cleaved RhoGDIβ was also observed under these conditions. Subcellular fractionation studies revealed the nuclear association of both full-length as well as cleaved forms of RhoGDIβ in β-cells under the duress of metabolic stress [39]. No significant impact on the association of RhoGDIβ with the nuclear fraction was seen in beta cells following acute exposure to a stimulatory concentration of glucose (conditions conducive for GSIS). In addition to RhoGDIβ, the nuclear association of RhoGDIα was also seen in β-cells under basal conditions, and hyperglycemic stress did not impact its abundance in the nucleus. Lastly, our data suggested little or no association of RhoGDIγ with the nuclear fraction isolated from cells exposed to either basal or hyperglycemic stress conditions. These findings have led us to conclude that the translocation of RhoGDIβ to the nuclear compartment in β-cells exposed to chronic hyperglycemic conditions might represent a key signaling step involved in the loss of β-cell function under metabolic stress [39]. Note that additional investigations in rodent and human islets are needed to further validate this postulation.

## 4. RhoGDIβ-Rac1 Signalome Contributes to Cellular Dysfunction

### 4.1. Evidence on Other Cells

Ota and coworkers have reported novel roles for the RhoGDIβ-Rac1 module in cancer cell progression [50]. The C-terminal truncated RhoGDIβ promoted metastasis through activating the Rac1 signaling pathway in ras-transformed fibroblast cells. Mechanism-based studies revealed that the C-terminal hydrophobic domain comprising Trp191, Leu193, and Ile195, which is requisite for interaction with isoprenyl groups of Rac1, is critical for the optimal functional regulation of Rac1. Selective deletion of the hydrophobic domain resulted in a constitutive association of RhoGDIβ with active GTP-bound Rac1. It was concluded that RhoGDIβ functions as a positive regulator of Rac1 due to its defective interaction with the isoprenyl groups of Rac1 [50].

Cho and associates investigated the mechanisms underlying RhoGDIβ-mediated effects on tumor cell survival and metastasis [51]. A significant upregulation of vascular endothelial growth factor C (VEGF-C) by RhoGDIβ in human gastric tumor tissues, as well as parental gastric cancer cell lines, was observed. The impact of RhoGDIβ in these cellular events was further validated by findings indicating a marked suppression of RhoGDIβ-mediated gastric cancer metastasis in cells following the specific depletion of VEGF-C. Furthermore, RhoGDIβ-mediated effects on VEGF-C expression involved a Rac1 activation step in that the suppression of Rac1 expression markedly attenuated RhoGDIβ-induced VEGF-C expression, leading to decreased invasiveness. These observations led to the conclusion that RhoGDIβ might serve as a therapeutic target to suppress metastasis risk, presumably involving a Rac1 activation step [51].

Kim and coworkers defined the roles of the RhoGDIβ-Rac1 module in tumor growth progression in gastric cancer [52]. Using yeast two-hybrid screening, they reported key regulatory roles for RhoGDIβ in promoting an interaction between Rac1 and Filamin A and the associated activation of Rac1 in the invasiveness of gastric cancer cells. A significant reduction in the migration and invasion ability of RhoGDIβ-expressing gastric cancer cells following the depletion of Filamin A was also observed. These studies have also identified Trio as the GEF for Rac1 activation in the invasive ability of gastric cancer cells. Together, these studies revealed critical roles for RhoGDIβ in promoting an interaction between Filamin-Rac1-Trio in exerting the invasive ability of gastric cancer cells [52].

Zhang and coworkers demonstrated the constitutive activation of Rac1 and its downstream signaling steps, including p38MAPK activation in MDA-MB-231 cells following the depletion of RhoGDIβ, suggesting the functional regulation of RhoGDIβ in the activation of Rac1 and its downstream signaling steps, including stress kinase activation. These findings further affirm the upstream regulatory role(s) for RhoGDIβ in the sustained activation of Rac1 [53].

It is noteworthy that, in addition to RhoGDIβ, RhoGDIγ has been shown to contribute to cell dysregulation via the modulation of smgs. For example, Zhang and coworkers reported the overexpression of RhoGDIγ in certain human breast cancer cell lines [54]. The transfection of these cells with siRNA-RhoGDIγ markedly reduced tumor growth and lung metastasis of highly invasive MDA-MB-231 breast cancer cells. RhoGDIγ was specifically associated with Rac1 and Rac3, but not other smgs, including Cdc42, RhoA, RhoC, and TC10, in these cells. The siRNA-mediated depletion of RhoGDIγ resulted in sustained activation of Rac1, leading to its translocation from the cytosol to the membrane for further activation of effector proteins, including p38 and JNK kinases. Lastly, they demonstrated that the inhibition of Rac1 suppresses p38/JNK kinase activities and the spontaneous anoikis of RhoGDIβ-depleted cells. It was proposed that the RhoGDIγ-Rac1 signaling module might play novel roles in breast cancer tumorigenesis [53,54]. In summary, increased crosstalk between RhoGDIs and smgs (e.g., Rac1) appears to contribute to cellular dysregulation induced by various stimuli.

### 4.2. Evidence in Pancreatic Beta Cells

As mentioned above, the activation of specific smgs (Arf6, Cdc42, Rac1, and Rab) is requisite for GSIS to occur. Briefly, Jayaram et al. demonstrated sequential activation, by glucose, of Arf6 (~1 min), Cdc42 (~3 min), and Rac1 (~15–20 min) in pancreatic beta cells [55]. Investigations from the Thurmond laboratory have validated the hypothesis that sequential activation of Cdc42 and Rac1 is requisite for GSIS [56]. Using a mouse model in which Rac1 is conditionally deleted in pancreatic beta cells, Asahara and coworkers validated the requisite roles of Rac1 in GSIS [57]. It should be noted that, in addition to the Rho subfamily of smgs (Rac1, Cdc42), the activation of Ras and Rab subfamily of G proteins is also critical for GSIS [17,18,58,59,60,61]. Together, these data demonstrate novel regulatory roles for smgs, including Rac1, in physiological insulin secretion.

Interestingly, evidence in multiple cell types, including the pancreatic beta cell, provided compelling support for the postulation that Rac1 is constitutively activated (sustained activation) under conditions of metabolic stress (chronic exposure to hyperglycemia, hyperlipidemia, and biologically active sphingolipids, such as ceramides) [62,63]. These in vitro observations in human and rodent islets, as well as clonal beta cells, have been validated in islets derived from animal models of impaired insulin secretion and diabetes, and in islets from human donors with T2D [62]. Complementary investigations in this field have suggested that the constitutive activation of Rac1 promotes the activation of oxidases (phagocyte-like NADPH oxidase; Nox2) and stress kinases (p38MAPK; JNK1/2), leading to increased oxidative stress and cell dysregulation and the loss of functional beta cell mass [62,64,65]. Importantly, in contrast to its expected translocation to the membrane following acute stimulation with glucose, the sustained activation of Rac1, seen under metabolic stress conditions, has been shown to result in its translocation to the nucleus to accelerate apoptotic events, such as the activation of p53 [66]. These findings have led to the suggestion that Rac1 plays both beneficial and detrimental roles in the islet beta cell function.

What are the potential mechanisms underlying the dual roles of Rac1 in normal islet function or dysfunction under metabolic stress? Existing evidence suggests that post-translational geranylgeranylation of Rac1 might contribute to its functional outcome. Briefly, acute exposure of the beta cell to an insulinotropic concentration of glucose results in the activation of protein prenyltransferases, leading to increased prenylation of Rac1 and its regulation of intracellular events (e.g., remodeling of actin cytoskeleton, translocation and fusion of secretory granules with the plasma membrane), culminating in insulin secretion. In contrast, the chronic exposure of islet β cells to gluco-lipotoxic and endoplasmic reticulum stress leads to the caspase-mediated degradation of the α-subunit of prenyl transferases, resulting in their functional inactivation. This, in turn, leads to the paradoxical activation and mistargeting of unprenylated Rac1 to the nuclear compartment, thus triggering apoptotic events, culminating in β-cell dysregulation and demise ([67]; see below for additional details).

Together, based on the above discussion, it appears that both Rac1 and its regulatory proteins (full and truncated forms of RhoGDIβ) translocate to the nuclear compartment in pancreatic beta cells under hyperglycemic stress. The potential impact of these events within specific subcellular sites (e.g., nucleus) remains to be addressed further. Interestingly, evidence on multiple cell types, including more recent observations in the islet beta cell, appears to suggest potential contributory roles for RhoGDIβ-Rac1 in the onset of cell dysfunction induced by a variety of stimuli. Some of these findings are reviewed in the next section. In addition, the next section is dedicated to highlighting novel roles of caspase recruitment domain-containing protein 9 (CARD9), a scaffolding protein, in the regulation of the RhoGDIβ-Rac1 signalome, leading to the genesis of beta cell dysfunction under metabolic stress.

### 4.3. Does CARD9 Contribute to RhoGDIβ-Rac1-Mediated Beta Cell Dysfunction Under Hyperglycemic Stress Conditions?

CARD9, a scaffolding protein, is widely expressed in macrophages and neutrophils [68,69,70,71,72,73]. It partakes in the transmission of signals via the plasma membrane-associated pattern recognition receptors to specific intracellular signaling sites. A growing body of evidence suggests key roles for CARD9 in the pathology of metabolic diseases, including insulin resistance and obesity [74]. Recent experimental evidence, albeit limited, suggests novel roles for CARD9 in the regulation of Rac1 activation. For example, Wu and coworkers demonstrated requisite roles for CARD9 for killing intracellular bacteria in macrophages. Mechanism-based experiments revealed the association/complexation of CARD9 with RhoGDIβ in phagosomes after bacterial and fungal infection. Furthermore, they demonstrated that the complexation of CARD9-RhoGDIβ relieved RhoGDIβ-induced inhibition of Rac1, leading to increased reactive oxygen species production and bacterial killing in macrophages [75]. These data implicate specific roles for CARD9 in the regulation of smg activation via its binding to RhoGDIβ, thus dissociating the RhoGDIβ-Rac1 complex.

**Figure 2:**
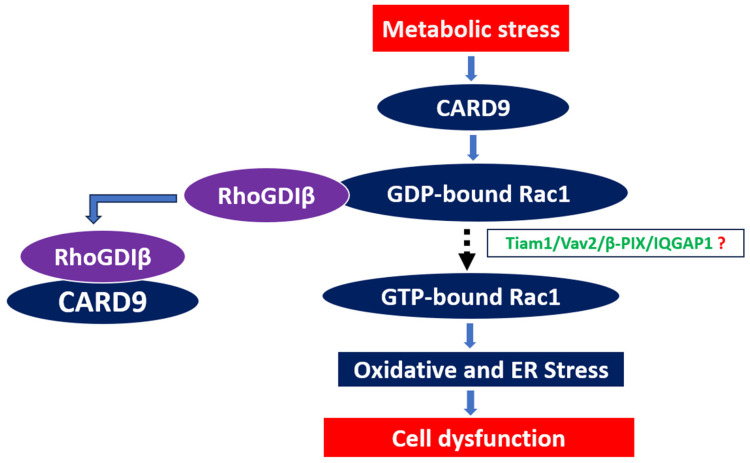
Proposed model for the involvement of CARD9-RhoGDIβ crosstalk in metabolic stress-induced beta cell dysfunction and demise. Based on our current knowledge, we propose that long-term exposure of pancreatic beta cells to metabolic stress conditions leads to the association of CARD9 with RhoGDIβ, thereby dissociating the RhoGDIβ–GDP-bound Rac1 complex. The “free” form of GDP-bound Rac1 is then activated by GEFs, which are relatively specific for Rac1. Published evidence suggests that Tiam1 and Vav2 might contribute to the activation of Rac1 by promoting GDP/GTP exchange. We propose that activation of Rac1 leads to activation of downstream stress kinases (p38MAPK, JNK1/2) and oxidases (Nox2), leading to increased intracellular stress culminating in beta cell dysregulation and demise (see text for additional details). Experimentally validated steps in this schematic are represented by bold arrows, whereas the proposed (hypothetical) steps are indicated by dotted arrows. Abbreviations: CARD9: caspase recruitment domain-containing protein 9; ER stress: endoplasmic reticulum stress; GDP-bound Rac1: inactive form of Rac1; Rac1-GTP: active form of Rac1.

In this context, we recently reported that CARD9 is expressed in insulin-secreting INS-1 832/13 cells, mouse islets, rat islets, and human islets [76]. Subsequent investigations have examined the critical roles of CARD9 in the genesis of islet beta cell dysfunction under chronic hyperglycemic stress. Our findings suggested a significant increase in the expression of CARD9 in insulin-secreting INS-1 832/13 cells following exposure to hyperglycemic conditions. The siRNA-mediated depletion of CARD9 expression markedly suppressed high-glucose-induced (constitutive) activation of Rac1 and its downstream stress kinase (p38MAPK) activation in these cells, suggesting novel roles for CARD9 in the metabolic dysregulation of the islet beta cell [77]. In a manner akin to the observations of Wu and coworkers [75], coimmunoprecipitation experiments indicated the inhibition of interaction between RhoGDIβ and Rac1 in INS-1 832/13 cells exposed to HG conditions. Together, our observations provided the first experimental evidence to indicate that HG conditions promote a significant increase in the interaction between CARD9 and RhoGDIβ, resulting in dissociation of the RhoGDIβ-Rac1 complex to enable the subsequent activation of Rac1 by candidate GEFs [77]. Indeed, evidence from the studies of Groysman et al. on the interaction between RhoGDIβ and Vav proteins (known GEFs for Rac1) provides additional support for the crosstalk between GEFs and RhoGDIβ in the regulation of cell function [78,79]. Additional studies are needed to precisely identify the putative GEFs that might mediate the activation of Rac1 (see below). Based on the information available to date, we propose that exposure of beta cells to chronic metabolic stress conditions leads to complexation/association between RhoGDIβ and CARD9, thereby releasing Rac1 for subsequent activation by GEFs (Figure 2). We also propose that RhoGDIβ might contribute to the G protein-mediated propagation of appropriate apoptotic signals from the soluble (cytosolic) compartment to the nucleus, thus promoting cellular dysfunction and demise (Figure 2). In addition to the model proposed in Figure 2, CARD9 might induce beta cell dysregulation via the BCL10-Rac1 signalome [80].

### 4.4. Evidence Affirming Novel Roles of CARD9 in the Induction of Metabolic Dysfunction in Animal Models of Obesity, Glucose Intolerance, and Insulin Resistance

In addition to its originally proposed roles in innate immunity, available evidence suggests that CARD9 plays novel regulatory roles in the development of glucose intolerance and insulin resistance in animal models of diet-induced obesity, glucose intolerance, and insulin resistance. For example, using a CARD9 knockout animal model, Zeng and coworkers investigated the roles of this scaffolding protein in high-fat diet (HFD)-induced obesity and associated metabolic complications [81]. Their findings suggested a significant reduction in HFD-induced insulin resistance and glucose intolerance in the CARD9 knockout mice compared to the wild-type mice. HFD treatment markedly induced the expression of p38 MAPK, JNK, and ERK in the wild-type mice without exerting any noticeable effects in the CARD9-depleted mice. It was concluded that the deletion of CARD9 might afford protection against metabolic dysfunction associated with diet-induced obesity, presumably mediated via the CARD9-MAPK signaling axis. Along these lines, Cao and coworkers investigated the regulatory roles of CARD9 in HFD-induced and obesity-associated myocardial dysregulation in these animals [82]. A significant increase in the expression of CARD9 in macrophages and heart tissue was observed in HFD-fed animals. A marked reduction in HFD-induced insulin resistance and glucose intolerance was observed in the CARD9 knockout mouse model. The deletion of CARD9 also afforded protection against obesity-induced myocardial dysregulation, interstitial fibrosis, and the infiltration of macrophages into the heart. Mechanism-based studies demonstrated significant protection of HFD-induced p38MAPK phosphorylation and pro-inflammatory cytokine production in CARD9 knockout animals. Lastly, CARD9 deletion restored the dysfunctional myocardial autophagy associated with HFD-induced obesity. Based on these findings, it was surmised that CARD9 deletion affords protection against HFD-induced inflammation, insulin resistance, metabolic abnormalities, and T2DM either by suppressing NF-κB and MAPKs signaling modules and/or improving insulin action and increased energy metabolism.

Despite the above evidence of the critical roles of CARD9 in the onset of metabolic dysfunction in animal models of glucose intolerance and insulin resistance, the putative roles of this adaptor protein in islet beta cell dysfunction leading to metabolic dysfunction in animal models remain understudied. However, as stated above, recent in vitro evidence from our laboratory has suggested significant similarities in the signaling modules that appear to be controlled by CARD9 in pancreatic beta cells under metabolic stress [77]. For example, we noted a significant increase in CARD9 expression in INS-1 832/13 cells and mouse islets exposed to metabolic stress conditions. The siRNA-mediated knockdown of CARD9 significantly attenuated high-glucose-induced activation of Rac1, the phosphorylation of p38MAPK and p65 subunit of NF-κB, and the expression of CHOP (a marker for ER stress). Complementary pharmacological investigations involving EHT-1864, a specific inhibitor of Rac1, have revealed a significant inhibition of high-glucose-induced p65 phosphorylation and nuclear association of STAT3. Based on these data, we concluded that CARD9 regulates the activation of the Rac1-p38MAPK-NFκB signaling pathway, leading to functional abnormalities in beta cells under metabolic stress conditions [83]. Together, these observations further implicate CARD9 as a mediator of metabolic stress-induced dysfunction of the islet beta cell. Future studies will validate these findings in human islets. It may be germane to point out that, using a dual systems genetic approach, Kaur and coworkers have recently identified CARD9 as one of the nine genes identified in the “T1D-T2D islet expression quantitative trait locus interaction network” in human islets. Additional studies are warranted to precisely identify the roles of these genes (e.g., CARD9) in islet beta cell function in health and diabetes.

Lastly, the above observations on CARD9′s role in islet β-cell dysfunction might provide additional mechanistic insights into how inflammation or immune cell crosstalk with β-cells plays critical regulatory roles in the onset of β-cell dysfunction. For example, Cosentino and Regazzi highlighted the potential contributory roles of macrophages in the initiation of autoimmune insulitis and low-grade chronic inflammation in the pathogenesis of T1DM and T2DM, respectively [84]. Likewise, Chen and associates [85] and Akhtar and coworkers [86] described the putative mechanisms involving various inflammatory signalomes that might contribute to the onset of T2DM. Indeed, a better understanding of putative inflammatory signaling pathways and their interaction with islet beta cells is critical for the development of therapeutic modalities to prevent the onset of both T1DM and T2DM.

## 5. Conclusions and Potential Knowledge Gaps and Opportunities for Future Research

**Figure 3:**
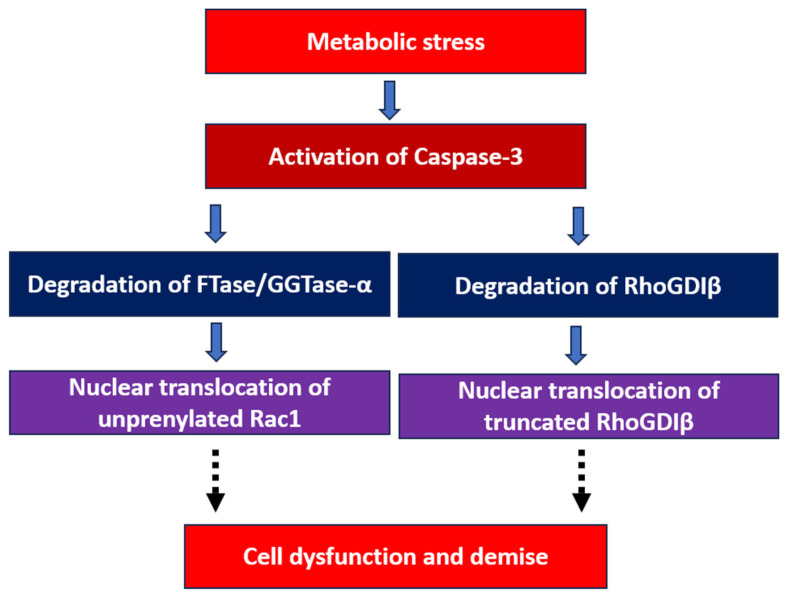
The proposed model for metabolic stress-induced nuclear translocation of Rac1 and the truncated RhoGDIβ and the onset of beta cell dysfunction and demise. Based on the existing information, we propose that metabolic stress conditions promote activation of caspase-3, leading to the degradation of several signaling proteins, including the common α-subunit of FTase/GGTase-1 (left arm of the model). Published evidence also suggests that the caspase-3-mediated degradation of FTase/GGTase-α results in the inactivation of FTase and GGTase, leading to inhibition of key smgs, including Rac1. Data in INS-1 832/13 cells, rat islets, and human islets suggest that the unprenylated but paradoxically active form of Rac1 translocates to the nuclear compartment. Furthermore, as indicated in the right arm of the model, caspase-3 activation leads to the increased degradation of RhoGDIβ, followed by the translocation of the degradation product of RhoGDIβ to the nuclear fraction. While it remains to be tested experimentally, we propose that the nuclear association of unprenylated but active Rac1 and the degradation product of RhoGDIβ singly or in combination would propagate the signaling steps leading to beta cell dysfunction and demise. Experimentally validated steps in this schematic are represented by bold arrows, whereas the proposed (hypothetical) steps are indicated by dotted arrows. Abbreviations: FTase/GGTase-α; common α-subunit of farnesyl and geranylgeranyl transferase-I.

Based on the above discussion and other relevant observations from extant studies, we propose (Figure 3) that metabolic stress conditions promote the activation of caspase-3, leading to the degradation of several signaling proteins, including the common α-subunit of FTase/GGTase-1 (left arm of the model). The caspase-3-mediated degradation of FTase/GGTase-α results in the inactivation of FTase and GGTase, leading to the inhibition of prenylation smgs, such as Rac1 [87]. Data on INS-1 832/13 cells, rat islets, and human islets suggest that the unprenylated but paradoxically active form of Rac1 (GTP-bound Rac1) translocates to the nuclear compartment [66]. Furthermore, as indicated in the right arm of the model, caspase-3 activation leads to the increased degradation of RhoGDIβ, followed by the translocation of the truncated product of RhoGDIβ to the nuclear fraction. While it remains to be validated experimentally, we propose that the nuclear association of unprenylated and active Rac1 (Rac1-GTP) and the degradation product of RhoGDIβ singly or in combination might accelerate the apoptotic signaling steps leading to beta cell dysfunction and demise under the duress of metabolic stress (Figure 3).

In conclusion, the above observations and postulations provide opportunities for future investigations in this field to further validate the roles of the RhoGDIβ-Rac1-CARD9 signaling module in the pathogenesis of islet dysfunction under metabolic stress conditions. It is our hope that future advances in this fertile area will help not only in our current understanding of this signalome in islet beta cell dysfunction but also in the development of small-molecule compounds (e.g., combinatorial chemistry approaches) with a high degree of specificity to inhibit this pathway to prevent beta cell dysfunction and demise under a variety of cellular stress conditions.

## Data Availability

Data will be made available upon request.

## Abbreviations

APPL2	Adaptor Protein–Phosphotyrosine Interacting with PH Domain and Leucine Zipper 2
ARF6	ADP ribosylation factor 6
ARNO	ARF nucleotide-binding site opener
β-PIX	Guanine nucleotide exchange factor for small G proteins (also known as ARHGEF7)
CARD9	Caspase recruitment domain-containing protein 9
Cdc42	Cell division control protein 42 homolog
Epac	Exchange protein directly activated by cAMP
GAP	GTPase-activating protein
GEF	Guanine nucleotide exchange factor
GDI	GDP dissociation inhibitor
GSIS	Glucose-stimulated insulin secretion
GPCR	G protein-coupled receptor
INS-1 832/13 cells	INS-1 832/13 rat insulinoma cell line
IQGAP	IQ motif containing GTPase-activating protein 1
JNK1/2	c-Jun N-terminal kinases 1 and 2
Nox2	Phagocyte-like NADPH oxidase
P38MAPK	p38 mitogen-activated protein kinase
PTMs	Post-translational modifications
Rac1	Ras-related C3 botulinum toxin substrate 1
RhoGDIα	Rho GDP dissociation inhibitor α
RhoGDIβ	Rho GDP dissociation inhibitor β
RhoGDIγ	Rho GDP dissociation inhibitor γ
Raf-1	Raf-1 proto-oncogene, serine/threonine kinase-1
RhoGDI	Rho GDP dissociation inhibitor
siRNA	Small interfering RNA
T2DM	Type 2 diabetes
Tiam1	T-cell lymphoma invasion and metastasis-inducing protein 1
Vav2	Vav guanine nucleotide exchange factor 2
